# An Unusual Case of Multiple Myeloma with Light-Chain Cast Nephropathy Secondary to a Very Large Plasmacytoma without Bone Marrow Involvement

**DOI:** 10.1155/2022/7531142

**Published:** 2022-02-21

**Authors:** Justin Komisarof, Jodi Lipof, Joseph DiTursi, Amit Chowdhry, Hae Yoon Grace Choung, W. Richard Burack, Louis Constine, Frank Passero

**Affiliations:** ^1^Department of Medicine, University of Rochester Medical Center, 601 Elmwood Avenue, Rochester, NY 14642, USA; ^2^Department of Hematology and Oncology, Wilmot Cancer Institute, 601 Elmwood Avenue, Rochester, NY 14642, USA; ^3^Department of Radiation Oncology, Wilmot Cancer Institute, 601 Elmwood Avenue, Rochester, NY 14642, USA; ^4^Department of Pathology and Laboratory Medicine, University of Rochester Medical Center, 601 Elmwood Avenue, Rochester, NY 14642, USA

## Abstract

Here, we report a case of a patient who presented to Strong Memorial Hospital with new-onset renal failure and anemia and was found to have multiple myeloma with lambda light-chain cast nephropathy secondary to a very large (14 cm × 14 cm × 12 cm) plasmacytoma without bone marrow involvement. This case is notable as solitary plasmacytomas are almost never seen with concomitant myeloma-defining CRAB criteria or significantly elevated serum free light-chain ratios. Although solitary plasmacytomas are typically definitively treated with radiation, this case highlights that systemic treatment may be helpful in certain clinical scenarios.

## 1. Introduction

Multiple myeloma is a plasma cell dyscrasia characterized by clonal plasma cells >10% on bone marrow biopsy or the presence of a plasmacytoma in the setting of clinical features known as “CRAB criteria,” which include hypercalcemia, renal insufficiency, anemia, or the presence of lytic bone lesions [1]. In the absence of CRAB criteria, a diagnosis of multiple myeloma can also be made by biomarker criteria including a free light chain ratio greater than 100, bone marrow plasma cell clonality greater than 60%, or multiple focal lesions on magnetic resonance imaging (MRI) [1]. Plasmacytomas are usually found within osseous structures but have been described in a wide range of extramedullary locations, including the liver, upper respiratory tract, gastrointestinal tract, soft tissues, and kidneys, among others [2–8]. A single plasmacytoma with minimal (<10%) or absent clonal marrow plasma cell involvement is a separate disease entity from multiple myeloma classified as a solitary plasmacytoma [9]. Solitary plasmacytomas are often treated with definitive radiation, although patients have a significant risk of progression to multiple myeloma at about 30% within 10 years of diagnosis [10–12]. The risk of progression is greater in patients with solitary plasmacytoma of bone than in patients with extramedullary plasmacytoma. Occasionally, solitary plasmacytomas may produce detectable and quantifiable monoclonal paraprotein that can be found on serum protein electrophoresis (SPEP), on serum immunofixation (IFE), or by an excess of kappa or lambda serum free light chains (sFLCs), resulting in an abnormal kappa-to-lambda ratio [13]. However, solitary plasmacytomas with low levels or undetectable disease in the bone marrow are very rarely reported with concomitant myeloma-defining CRAB criteria or a significantly elevated sFLC ratio. Here, we report a case of a patient who presented to Strong Memorial Hospital with new-onset renal failure and anemia and was found to have multiple myeloma with light-chain cast nephropathy secondary to a very large plasmacytoma without any other signs of end-organ damage or bone marrow plasma cell involvement.

## 2. Case Report

**Figure 1:**
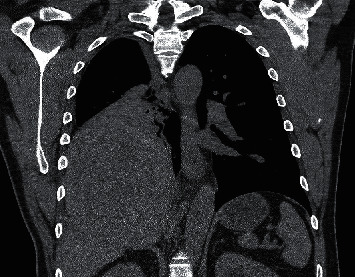
CT scan of the chest on presentation to hospital revealing very large (14 × 14 × 12 cm) lung mass.

A 57-year-old male with a past medical history significant for papillary thyroid cancer treated definitively with thyroidectomy presented to his primary care physician with progressive fatigue, headache, and abdominal cramping. He reported dramatically decreased urine output starting two days prior to presentation, despite aggressive hydration. Initial workup revealed a markedly elevated creatinine level at 4.68 mg/dL. The patient had no prior history of kidney disease. His last known creatinine level, obtained 8 months prior, was 1.04 mg/dL. Initial laboratory studies also revealed new mild anemia with a hemoglobin level of 11.1 g/dL. He was sent to the emergency department for further evaluation. Additional lab studies on presentation revealed worsening renal function with a creatinine level of 7.72 mg/dL and hyponatremia with sodium of 127 mmol/L and normal serum osmolality of 285 mOsm. The 24-hour urine protein level was 750 mg. Renal ultrasound showed no evidence of hydronephrosis and normal-sized kidneys (11.4 cm for the R kidney and 12.3 cm for the L kidney). A chest X-ray was obtained due to decreased right-sided breath sounds on exam, which showed a large right hemithorax opacification. Follow-up CT chest revealed a 14.0 × 14.0 × 12.0 cm mass in the lower lobe of the right lung (Figure 1) as well as an adjacent 1.1 cm lytic lesion in the proximal right 11^th^ rib. The patient denied any pulmonary symptoms.

**Figure 2:**
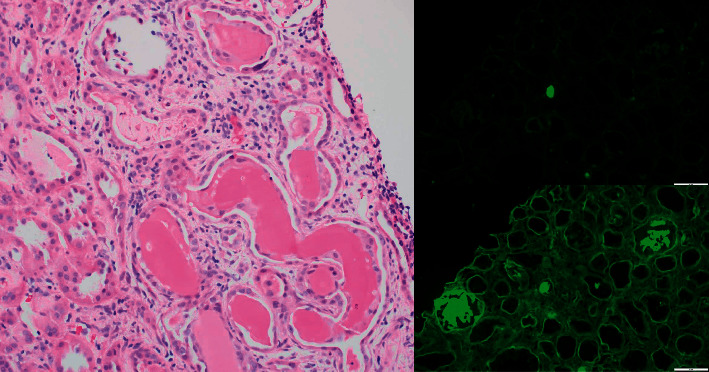
H&E stain of renal biopsy revealing atypical glassy eosinophilic casts associated with cellular reaction in acutely injured tubules (a). Immunofluorescence on renal biopsy is negative for kappa free light chains (b) but shows 2–3+ staining in the atypical casts for lambda free light chains (c).

**Figure 3:**
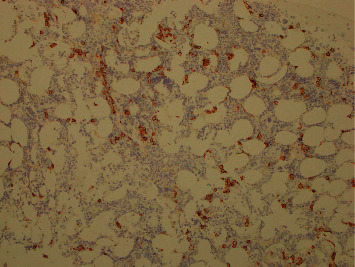
CD138 immunohistochemical stain (Giemsa background) of bone marrow biopsy showing scattered plasma cells in a physiologic distribution with no evidence of neoplasm.

**Figure 4:**
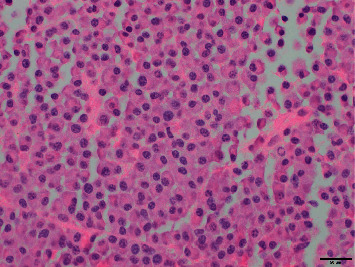
H&E stain of lung biopsy consistent with plasmacytoma.

The patient's kidney function continued to worsen, and despite receiving fluids, he remained oliguric, requiring initiation of emergent hemodialysis. He underwent a kidney biopsy that revealed light-chain cast nephropathy, lambda type, with diffuse acute tubular injury (Figure 2). This finding raised suspicion for multiple myeloma. An M spike was detected on SPEP with a quantifiable paraprotein level of 0.4 g/dL, and lambda light-chain monoclonal protein was detected on serum immunofixation. The serum lambda free light-chain (FLC) level was 776 mg/dL, with an involved (lambda)-to-uninvolved (kappa) sFLC ratio of 160.3. A skeletal survey showed no additional sites of osteolytic lesions. Bone marrow biopsy revealed a slight increase in plasma cells (5%) but did not show any evidence of clonality by flow cytometry or fluorescence in situ hybridization (FISH) (Figure 3). The patient was urgently started on cyclophosphamide (300 mg/m^2^ orally on days 1, 8, and 15), bortezomib (1.3 mg/m^2^ subcutaneously on days 1, 4, 8, and 11), and dexamethasone (40 mg orally on days 1, 4, 8, and 11) (CyBorD) chemotherapy as well as plasmapheresis. Biopsy of the lung mass was performed, and the pathology was consistent with plasmacytoma, with lambda-restricted clonal plasma cells (Figure 4). As there were no clonal plasma cells on the bone marrow sample to perform CD138-directed FISH, the sample from the lung plasmacytoma was sent for myeloma FISH analysis, and this revealed a plasma cell clone with 13q deletion and monosomy 14. The patient initially responded well to chemotherapy and four sessions of plasmapheresis, with a reduction in lambda FLC level to 87 mg/dl. He was discharged from the hospital to continue treatment as an outpatient.

In the outpatient setting, the patient achieved at least a very good partial response (VGPR) with continued chemotherapy. The patient's kidney function continued to improve, and he was able to discontinue dialysis within one month of initiation, although his kidney function did not recover to his previous baseline. Positron emission tomography/computed tomography (PET/CT) scan performed after one cycle of chemotherapy did not show any evidence of osseous disease or additional plasmacytomas. A repeat chest CT after 4 cycles of treatment demonstrated an interval decrease in the size of the plasmacytoma to 13.0 × 11.0 × 10.0 cm. Due to the development of mild neuropathy and a relative plateau in levels of FLC, his chemotherapy was switched to lenalidomide 10 mg orally (PO) for 21–28 days, bortezomib 1.3 mg/m^2^ weekly, and 40 mg of dexamethasone PO weekly. His lambda free light chains reached a nadir of 57 mg/dL but rose to 78 mg/dL on this regimen, and subcutaneous (SC) daratumumab 1800 mg weekly was added, which resulted in a marked decrease in his lambda FLC level to 5 mg/dL. He was referred to radiation oncology for treatment of the plasmacytoma and underwent external beam radiation therapy (EBRT) with 52.2 Gy delivered in 29 fractions. Following radiation, his lambda FLC completely normalized. His therapy was de-escalated to dexamethasone 40 mg PO monthly and daratumumab 1800 mg SC monthly. He has remained stable on this regimen with no biochemical evidence of recurrence at the time of this report. A repeat PET/CT showed a further decrease in the size of the mass, measuring approximately 8.8 cm × 6.7 cm without significant SUV uptake, consistent with treatment response.

## 3. Discussion

In this report, we describe a patient who presented to our institution with new-onset renal failure due to biopsy-proven light-chain cast nephropathy from multiple myeloma secondary to a very large plasmacytoma. It is not clear whether this plasmacytoma was extramedullary or whether it initially arose from the patient's right 11^th^ rib lytic lesion and invaded the lung. The rib lesion was not hypermetabolic on PET/CT, which has high sensitivity and specificity in this setting [14], and it is possible that the rib lesion was caused by compression from the adjacent tumor and was not a focus of myeloma at all. However, as the patient received systemic treatment prior to PET/CT, it is also possible that the rib may have lost the PET avidity that was originally present. Ultimately, without a biopsy of the rib lesion at the time of diagnosis, the plasmacytoma's origin cannot be definitively determined.

Regardless of the plasmacytoma's origin, this appears to be the first case reported of a large plasmacytoma without marrow involvement, fulfilling the International Myeloma Working Group definition of multiple myeloma [1] by the criteria of renal insufficiency secondary to biopsy-proven cast nephropathy. Although one prior case report described a patient with soft tissue plasmacytoma and myeloma cast nephropathy on renal biopsy, serum free light chains were not quantified in that case and a bone marrow biopsy was not performed prior to systemic therapy [15]. This presentation was similar to but also distinct from the rare phenomenon of macrofocal myeloma, in which multiple bone plasmacytomas are seen in the absence of bone marrow involvement. At this time, the standard of care for macrofocal multiple myeloma is systemic bortezomib-based therapy as a bridge to autologous stem cell transplant [16, 17]. These patients typically respond well to treatment and have a significantly increased overall median survival compared to standard myeloma patients [17].

Solitary plasmacytomas with little or no bone marrow involvement do not normally result in light-chain cast nephropathy. While many patients with solitary plasmacytoma have a detectable serum monoclonal protein, the median M spike was 0 g/dL and 0.5 g/dL in two case series [13, 18]. Approximately half of the patients had an abnormal sFLC ratio, but the median involved-to-uninvolved ratio was low at 2.3 [13, 18]. In this case, the large size of our patient's plasmacytoma likely represented a high burden of disease. Previous studies have shown that increased plasmacytoma size is correlated with a higher risk of progression to multiple myeloma [18–20] as well as relapse after radiotherapy [21]. This plasmacytoma was also associated with a significant serum paraprotein, which is independently a poor prognostic factor in extramedullary plasmacytoma [18]. Further investigation into a possible correlation between the size of a solitary plasmacytoma and existence of serum paraprotein could lead to a better understanding of this pathology and lead to a better ability to predict patient outcomes.

Typically, solitary plasmacytomas, with or without the presence of bone marrow involvement, can be treated definitively with radiotherapy [22, 23]. This patient was treated with systemic therapy immediately following the results of his kidney biopsy. At that time, his bone marrow biopsy had not yet resulted, and based on his serum studies with new anemia and renal failure requiring dialysis, it was presumed that he had moderate to high marrow involvement. After his bone marrow biopsy resulted, the decision was made to continue systemic therapy due to his clinically relevant reduction in lambda FLC and improving renal failure. Our patient also received plasmapheresis. The utility of plasmapheresis in patients with myeloma associated with acute renal failure remains unresolved by randomized clinical trials and has been called into question in the era of highly active proteasome inhibitor-based regimens [24, 25]. However, our patient had rapidly progressive renal failure requiring dialysis with a high serum free light-chain level. Thus, plasmapheresis was incorporated into his therapeutic plan to provide the highest likelihood of rapid serum free light reduction and renal recovery pending a more durable response from chemotherapy.

It is possible that the patient's disease could have been controlled with radiotherapy alone. However, recent studies have found a significant benefit to systemic therapy for solitary plasmacytoma, with significantly lower rates of progression to multiple myeloma [26, 27]. Furthermore, in this case, due to the extreme size of the mass, initiating treatment with systemic therapy resulted in the shrinking of the tumor and thus reduction in the area requiring radiation. Further research is needed to determine optimal treatment strategies in patients with very large plasmacytomas that lack significant bone marrow involvement by clonal plasma cells but meet criteria for multiple myeloma based on laboratory findings.

## Data Availability

The imaging, laboratory, and pathologic data used to support the findings of this case study are included within the article.
